# Study of the Link Between Neuronal Death, Glial Response, and MAPK Pathway in Old Parkinsonian Mice

**DOI:** 10.3389/fnagi.2020.00214

**Published:** 2020-07-29

**Authors:** Ana Luisa Gil-Martinez, Lorena Cuenca-Bermejo, Pablo Gallo-Soljancic, Consuelo Sanchez-Rodrigo, Virginia Izura, Harry W. M. Steinbusch, Emiliano Fernandez-Villalba, Maria Trinidad Herrero

**Affiliations:** ^1^Clinical and Experimental Neuroscience Group (NiCE), Institute for Aging Research, School of Medicine, Regional Campus of International Excellence “Campus Mare Nostrum”, University of Murcia, Murcia, Spain; ^2^Biomedical Research Institute of Murcia (IMIB-Arrixaca), University of Murcia, Murcia, Spain; ^3^School for Mental Health and Neuroscience (MHeNs), Department of Psychiatry and Neuropsychology, Maastricht University, Maastricht, Netherlands

**Keywords:** MAPKs, Parkinsonism, aging, neuroinflammation, neurodegeneration

## Abstract

**Background**: Parkinson’s disease (PD) is described as an age-related neurodegenerative disorder. However, the vast majority of research is carried out using experimental models of young animals lacking the implications of the decline processes associated with aging. It has been suggested that several molecular pathways are involved in the perpetuation of the degeneration and the neuroinflammation in PD. Among others, mitogen-activated protein kinases (MAPKs) have been highly implicated in the development of PD, and regulating components of their activity are indicated as promising therapeutic targets.

**Methods**: To further define how MAPKs expression is related to the glial response and neuronal cell death, Parkinsonism was induced under an acute regimen in old mice. Moreover, the sacrifice was carried out at different time points (4, 8, 24, and 48 h) after 1-methyl-4-phenyl-1,2,3,6-tetrahydropyridine hydrochloride (MPTP) injections to describe the early dynamic changes over time produced by the intoxication.

**Results**: The results revealed that neuronal death increases as glial response increases in the nigrostriatal pathway. It was observed that both processes increase from 4 h in the ventral mesencephalon (VM), and neuronal death becomes significant at 48 h. In the striatum, they were significantly increased from 48 h after the MPTP administration compared with that in the control mice. Moreover, the p-ERK levels decrease, while phospho-p38 expression increases specifically in the striatum at 48 h after MPTP intoxication.

**Conclusions**: The importance of these data lies in the possibility of elucidating the underlying mechanisms of neurodegenerative processes under aging conditions to provide knowledge for the search of solutions that slow down the progression of PD.

## Introduction

Parkinson’s disease (PD) is the second most common neurodegenerative disorder in the world, affecting 100–200 per 100,000 people over 65 years old (Tysnes and Storstein, 2017). Its main pathological feature is the chronic and progressive loss of dopaminergic neurons within the substantia nigra pars compacta (SNpc). This leads towards a decrease in dopamine levels in the nigrostriatal pathway (Kastner et al., 1994) and in addition, to proteinaceous inclusions known as Lewy bodies (Cuenca et al., 2019).

Despite the efforts and the advances in the knowledge of PD, its development is mostly idiopathic and is still unknown. Specifically, sustained inflammatory responses may not be the first direct cause of the disease, but emerging evidence has shown that these contribute and perpetuate the initial neurodegenerative processes, with a persistent activation of the microglia which maintain phagocytic features even years after the insult (Halliday and Stevens, 2011; Barcia et al., 2013). Moreover, several studies suggest that the complex interactions between the deleterious mechanisms of aging involved levels of chronic mild inflammation in the SNpc, producing those dopaminergic neurons that become more vulnerable to degeneration (Calabrese et al., 2018).

It has been described that several inflammatory mediators can induce the deregulation of different mediating pathways of inflammation in experimental animal models of PD (Barcia et al., 2005, 2011). Among others, the mitogen-activated protein kinase (MAPK) signaling pathway is reported to possibly differentially contribute to the dopaminergic neuronal decline as observed in PD (Bohush et al., 2018). Moreover, the use of regulatory compounds of their activity has been related to beneficial effects (Gil-Martínez et al., 2018a). MAPKs are serine–threonine protein kinases that allow cells to respond to stimuli from their extracellular environment.

In mammals, the main proteins, part of the MAPK signaling pathway, are c-Jun NH_2_-terminal kinase (JNK), extracellular signal-regulated kinase (ERK), and p38 MAPK which contribute differentially to the dopaminergic neuronal degeneration characteristic of PD (Bohush et al., 2018). ERK, JNK, and p38 have a central role in the response to endogenous and environmental stress signals by regulating both cell survival and cell death induction (Kim and Choi, 2010). Specifically, it has been reported that TGF-β selectively inhibits pro-inflammatory cytokines through the crosstalk between p38 and ERK (Xiao et al., 2002). On the one hand, activation of the ERK signaling pathway has been described to mediate cell proliferation and differentiation (Plotnikov et al., 2011), although growing evidence also suggests its implication in neuronal death (Stanciu et al., 2000). On the other hand, the p38 MAPKs are required for the induction of cell death under different stimuli, mainly from extracellular stresses and cytokines (Corrêa and Eales, 2012). In this line, it is described that environmental toxins, like rotenone, can directly activate microglia (Gao et al., 2013) and increase the levels of reactive oxygen species (Wu et al., 2013), propitiating dopaminergic degeneration through the p38 MAPK and JNK pathway (Gil-Martínez et al., 2018b).

These facts altogether highlight the importance of investigating the dynamic changes over time of dopaminergic neuronal degeneration, astrocytic response, and its relations with the activation of p38 and ERK in old Parkinsonian mice.

## Materials and Methods

### Animals

The study was performed on 40 male C57BL/6N mice (20 months of age, weight 26–28 g) purchased from Charles River Laboratories (Charles River Laboratories Inc., Barcelona, Spain) and maintained in a room with regulated temperature (20 ± 2°C) and 12-h light/dark cycles. All *in vivo* experiments were performed according to the European Directive 2010/63/UE and the Spanish RD/53/2013 for the protection of animals used for experimentation and other scientific purposes. All the experimental methods and procedures were approved by the Institutional Committee on Animal Ethics of the University of Murcia (REGA ES300305440012).

### Experimental Groups and MPTP Intoxication Regime

**Figure 1:**
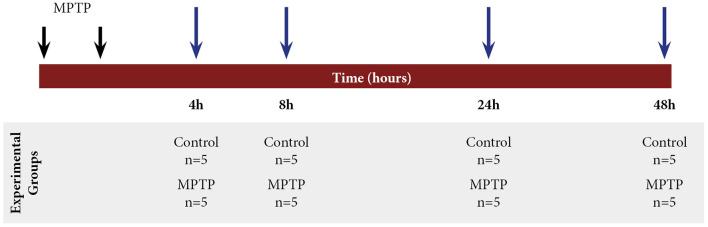
Scheme of the experimental design with mice distribution and different time points of sacrifice after 1-methyl-4-phenyl-1,2,3,6-tetrahydropyridine hydrochloride (MPTP) intoxication: 4, 8, 24, and 48 h.

All animals were randomly distributed into two main groups: control group (*n* = 20) and 1-methyl-4-phenyl-1,2,3,6-tetrahydropyridine hydrochloride (MPTP) group (*n* = 20). Parkinsonism was induced by intraperitoneal injections of MPTP (Sigma–Aldrich; 30 mg/kg/dose, two doses, 2-h interval) dissolved in saline (Annese et al., 2015). The control animals were treated with vehicle (saline) following the same protocol of administration of MPTP. The animals were sacrificed at different time points after MPTP intoxication: 4, 8, 24, and 48 h. The brains were removed, immediately dissected according to the areas of interest (ventral mesencephalon (VM) and striatum), and stored in −80°C until further processing (Figure 1).

### Sample Preparation

Approximately 40–90 mg of sample was homogenized in the proportion of 1 g tissue in 9 ml of lysis buffer (100 mM Tris-HCl, 200 mM NaCl, 1 mM EDTA, 2 mM DTT, 0.05% triton, and one tablet complete protease inhibitor mix/20 ml buffer) and one tablet PhosSTOP phosphatase inhibitor cocktail/10 ml buffer using a mini-Bead-Beater (BioSpec Products, Bartlesville, OK, USA). The samples were homogenized three times for 30 s, with 5 min of cooling on ice between runs. After 30 min of cooling on ice, these were stored at −80°C until further use. Protein quantification was performed with the Bio-Rad DC protein assay (Bio-Rad Laboratories, Inc., Hercules, CA, USA).

### Western Blot

The brain homogenates were separated with sodium dodecyl sulfate-polyacrylamide gel electrophoresis. The proteins were transferred onto a nitrocellulose membrane and blocked [1:1 Odyssey blocking buffer in phosphate-buffered saline (PBS) 1×, Li-Cor, Lincoln, NE, USA] for 1 h at room temperature. The membranes were incubated with the corresponding primary antibody overnight at 4°C (Table 1). The membranes were washed with PBS and PBS-Tween and incubated with secondary antibody for 1 h at room temperature, 1:10,000 goat anti-mouse IRDye 700 (#925-68070, Li-Cor), and/or 1:10,000 goat anti-rabbit IRDye 800 (#926-32211, Li-Cor). We attempted to use separate fluorescently labeled antibodies for GAPDH, TH, and GFAP, but we lost sensitivity with others, that is why we finally decided to use the ones shown in Table 1. The membranes were washed in PBS and PBS-Tween, and fluorescent protein bans were visualized using the Odyssey Infrared Imaging System (Li-Cor). Image Studio Lite was used to quantify the fluorescent protein bands.

**Table 1:** Dilutions for primary and secondary antibodies used for western blot.

Primary antibodies, code	Host	Dilution, incubation time	Secondary antibodies, code	Dilution, incubation time
Anti-TH	Mouse, MAB318, Millipore	1:500, o.v.	Goat anti-rabbit; Alexa 700	1:10,000, 1 h
Anti-GFAP	Mouse, MAB360, Millipore	1:500, o.v.	Goat anti-mouse; Alexa 700	1:10,000, 1 h
Anti-phospho-p38 MAPK (Thr180/Tyr182)	Rabbit, #4511, Cell Signaling Technology	1:1,000, o.v.	Goat anti-rabbit; Alexa 800	1:10,000, 1 h
Anti-phospho-p44/42 (Erk1/2; Thr202/Tyr204)	Rabbit, #4370, Cell Signaling Technology	1:2,000, o.v.	Goat anti-rabbit; Alexa 800	1:10,000, 1 h
Anti-GADPH	Mouse, AB9684, Abcam	1:1,000, o.v.	Goat anti-mouse; Alexa 700	1:10,000, 1 h

### Data and Statistical Analysis

Data are presented as mean ± SD and were analyzed by means of a two-way analysis of variance test (two-way ANOVA), with a Sidak *post hoc* analysis for multiple-group comparisons. The null hypothesis was rejected at a significance level of 0.05 so that differences with a value of *p* < 0.05 were considered as significant and those with a value of *p* < 0.01 were considered as statistically very significant. All statistical analyses were conducted using GraphPrism7 software (GraphPad Software Inc.).

### Sample Preparation and Immunohistochemical Procedure

After the animals were sacrificed, the brains were extracted and fixed for 12 h at 4°C in 4% paraformaldehyde. Then, they were washed with ethanol and embedded in paraffin blocks. The brain sections (5 μm) of SNpc and striatum were cut using Thermo Scientific HM 325 Rotary Microtome, Thermo Fisher Scientific, Waltham, MA, USA. The brain sections were immersed in xylene, in a gradient of ethanol (100, 95, and 80%), and distilled water. Antigenic retrieval was performed in citrate buffer for 30 min at 95°C (10 mM citric acid, pH 6.0). A solution of 0.3% H_2_O_2_ was used for 20 min to inhibit endogenous peroxidase, and non-specific bindings were blocked using a solution of tris-buffered saline (TBS) 0.1 M pH 8.4, 10% goat serum, and 0.5% Triton X-100. Then, the samples were incubated with primary antibodies, diluted in TBS + Tween 0.5% + 1% goat serum, and stored overnight at 4°C with mouse anti-TH (1:500, Millipore), rabbit anti-Iba1 (1:1,000, Millipore), and mouse anti-GFAP (1:500, Millipore). After washing with TBS, the sections were incubated with their corresponding secondary antibodies for 1 h (mouse anti-IgG, 1:250 and rabbit anti-IgG, 1:250; Vector Labs). Finally, the sections were washed, incubated with avidin/biotin conjugated to peroxidase (ABC Elite Kit, Vector Labs), and stained using 3,3′-diaminobenzidine Peroxidase HRP Substrate Kit (Vector Labs). Representative photomicrographs were taken using Hall 100 ZEISS optical microscope with an Axiocam ZEISS digital camera.

## Results

### Dopaminergic Neuronal Death in the VM and in the Striatum

**Figure 2:**
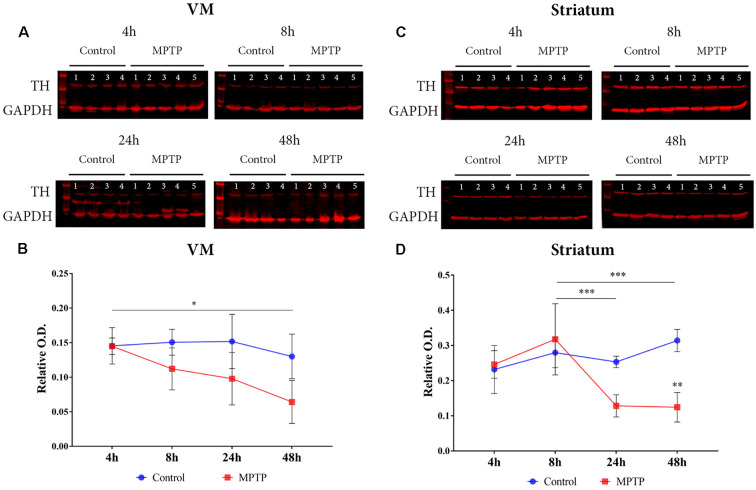
Dopaminergic neuronal death. **(A)** Western blot of TH expression in the ventral mesencephalon (VM). **(B)** Quantification by western blot of TH protein expression showed a significant decrease (*p = 0.014) in Parkinsonian animals sacrificed after 48 h compared to those sacrificed after 4 h. **(C)** Western blot of TH expression in the striatum. **(D)** Quantification by western blot showed a significant decrease of TH expression in Parkinsonian animals sacrificed after 24 h (***p < 0.001) and 48 h (***p < 0.001) compared to those sacrificed after 8 h.

The expression of TH was measured in both the VM and the striatum (Figure 2). A two-way ANOVA (Parkinsonism × time) was carried out, and the obtained results showed a significant main effect for Parkinsonism (*F*_(1,25)_ = 3.819, *p* = 0.022) and time (*F*_(3,25)_ = 13.760, *p* = 0.0010). There was no significant interaction between these two variables (*F*_(3,25)_ = 1.813, *p* = 0.171). The *post hoc* analysis showed a significant decrease (*p* = 0.014) of TH expression in Parkinsonian animals sacrificed at 48 h compared to the group sacrificed at 4 h in the VM.

In the striatum, there was a significant interaction for Parkinsonism × time (*F*_(3,24)_ = 7.753, *p* < 0.001) and a significant main effect for both variables Parkinsonism (*F*_(3,24)_ = 5.557, *p* = 0.005) and time (*F*_(1,24)_ = 11.15, *p* = 0.003). The comparison between groups showed a significant decrease in groups sacrificed at 24 h (*p* < 0.001) and at 48 h (*p* < 0.001) compared to the animals sacrificed at 8 h (Figures 2C,D).

### Astroglial Expression

**Figure 3:**
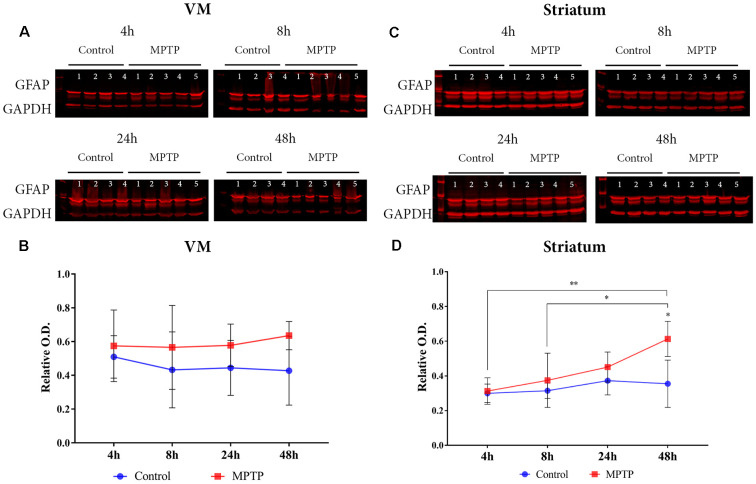
Astrocyte expression. **(A)** Western blot of GFAP in the VM. **(B)** Quantification by western blot of GFAP protein expression did not show a significant increase but a positive tendency in the Parkinsonian animals compared to the non-intoxicated mice can be observed. **(C)** Western blot of GFAP in the striatum. **(D)** Quantification by western blot showed a significant increase of GFAP expression in Parkinsonian animals sacrificed after 48 h (*p = 0.0370) compared with its control group and with 1-methyl-4-phenyl-1,2,3,6-tetrahydropyridine hydrochloride (MPTP) mice sacrificed after 4 h (**p = 0.0046) and 8 h (*p = 0.0473).

Astroglial activation was studied by GFAP expression in the VM (Figures 3A,B). The two-way ANOVA for Parkinsonism × time did not show a significant main effect (*F*_(3,25)_ = 0.586, *p* = 0.629) neither for time (*F*_(3,25)_ = 0.586, *p* = 1.377) nor for Parkinsonism (*F*_(1,25)_ = 0.288, *p* = 0.596; Figures 3A,B).

However, the results obtained from the two-way ANOVA analysis for the striatum showed a significant main effect for time (*F*_(3,26)_ = 4.684, *p* = 0.009) and for Parkinsonism (*F*_(3,26)_ = 8.377, *p* = 0.0076). No significant interaction (Parkinsonism × Time) was observed for both variables (*F*_(3,26)_ = 2.219, *p* = 0.109). The *post hoc* analysis showed a significant increase of GFAP expression in the group sacrificed at 48 h after MPTP intoxication compared with its control group (*p* = 0.0370) and with those Parkinsonian animals sacrificed after 4 h (*p* = 0.0046) and 8 h (*p* = 0.0473; Figures 3C,D).

### MAPK Activation in the Nigrostriatal System

**Figure 4:**
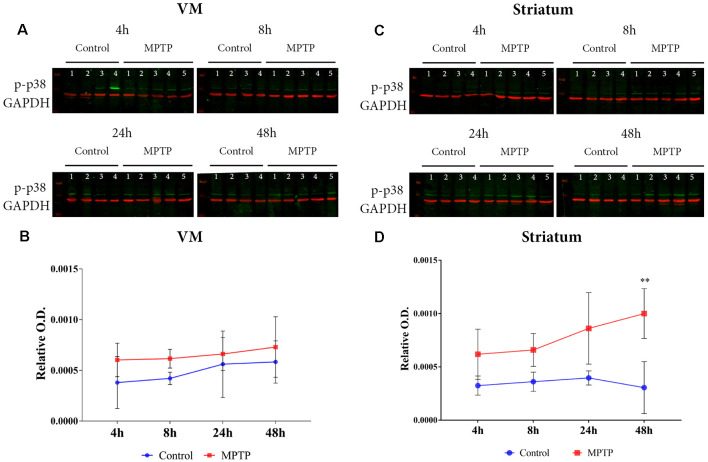
**(A)** Western blot of phosphor-p38 in the VM. **(B)** Quantification by western blot of phospho-p38 mitogen-activated protein kinase (MAPK) expression shows higher levels in 1-methyl-4-phenyl-1,2,3,6-tetrahydropyridine hydrochloride (MPTP) mice compared with control animals. **(C)** Western blot phosphor-p38 in the striatum. **(D)** Quantification by western blot showed a significant increase of phospho-p38 MAPK expression in Parkinsonian animals sacrificed after 48 h (**p < 0.0001) compared with its control group.

**Figure 5:**
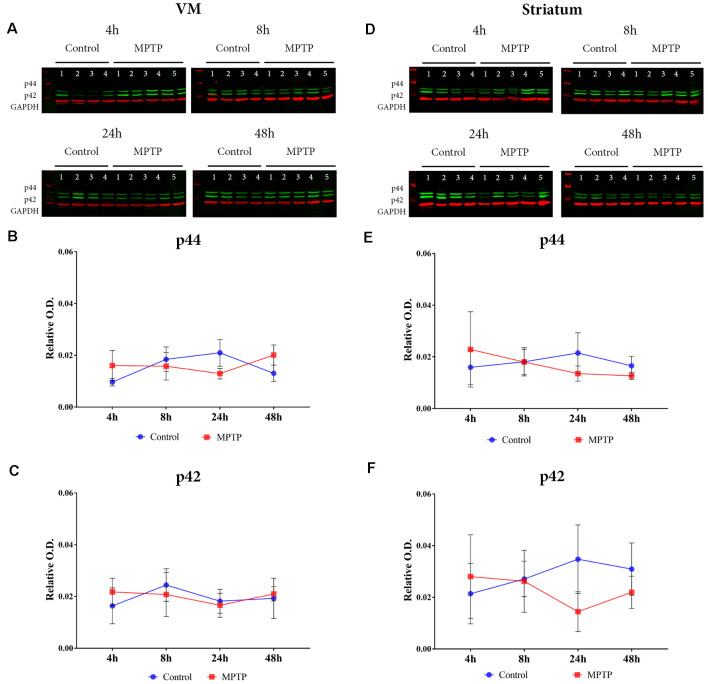
**(A)** Western blot phospho-p44/42 (Erk1/2) in the VM. **(B,C)** Quantification by western blot of phospho-p44 and phosphor-p42 protein expression did not show significant differences. **(D)** Western blot phospho-p44/42 (Erk1/2) in the striatum. **(E,F)** Quantification by western blot of phospho-p44 and phosphor-p42 protein expression did not show significant differences.

The results obtained for phospho-p38 MAPK in the VM showed a significant main effect for Parkinsonism (*F*_(1,27)_ = 6.171, *p* = 0.0195), but no significant effect was obtained for time (*F*_(3,27)_ = 1.139, *p* = 0.3512) and Parkinsonism × time (*F*_(3,27)_ = 1.139, *p* = 0.1356; Figures 4A,B). This means that, as can be observed in Figure 4B, the levels of phospho-p38 MAPK are mainly higher in all MPTP groups compared with those of the non-intoxicated mice, and they remain throughout the 48 h. In the striatum (Figures 4C,D), the expression levels of phospho-p38 MAPK begin with the same trend as in the VM at 4 and 8 h. However, after 24 h, there is an increase in the expression of phospho-p38 MAPK, which is highly significant at 48 h compared with its control group (*p* = 0.0012; Figure 4D). Importantly, as can be observed in Figure 5A, the VM panel at 4 h depicts a high p-p38 signal in control mouse 4 that we considered as an outlier due to a technical error in processing that specific sample. The two-way ANOVA showed a very significant main effect Parkinsonism (*F*_(1,27)_ = 36.01, *p* < 0.0001). Finally, the results obtained from the activation of ERK did not show significant changes both in the VM and in the striatum (Figure 5).

### Immunohistochemical Analysis for Dopaminergic Neuronal Death and Glial Activation

**Figure 6:**
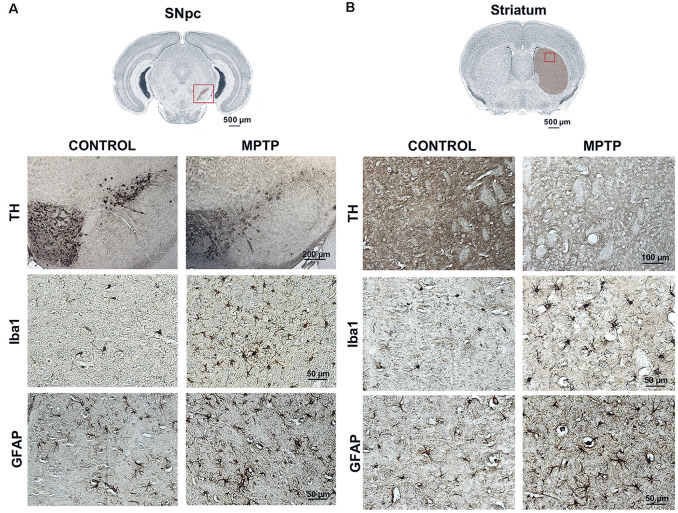
Representative images of TH, Iba-1, and GFAP immunostaining of control and Parkinsonian mice at 48 h after the last 1-methyl-4-phenyl-1,2,3,6-tetrahydropyridine hydrochloride (MPTP) injection. The microphotographs show a decrease in the number of TH-positive neurons and striatal fibers in the MPTP mice as well as an increase in the number of microglial and astroglial cells, with morphological changes. These findings are consistent with the data obtained in the western blot for dopaminergic neuronal death and astroglial response (magnification, ×40; scale bar = 50 μm, except for the figures of TH in the SNpc; magnification, ×10; scale bar = 200 μm and TH in the striatum; magnification, ×20; scale bar = 100 μm). **(A)** Coronal sections in the SNpc (scale bar = 500 μm). **(B)** Coronal sections in the striatum (scale bar = 500 μm).

Immunohistochemical analyses were performed to observe the dopaminergic neuronal loss, astroglial response, and microglia activation (by TH, GFAP, and Iba-1 immunostaining, respectively) both in control and MPTP mice at 48 h after the MPTP intoxication. In Figure 6, a decrease of TH-positive neurons in the SNpc and the TH-positive striatal terminals in the MPTP-treated mice can be observed compared with that in control mice. These observations are in line with the results obtained for TH in western blot at 48 h. Regarding neuroinflammatory markers, an increase of Iba-1 and GFAP-positive cells both in the SNpc and striatum of Parkinsonian mice can be seen compared with that in control animals. In addition, morphological changes for both types of cells are notable as well, including increment of cell body size and branches. These observations are in concordance with the results obtained in the western blot analysis for astroglial expression in MPTP mice at 48 h.

## Discussion

In the present study, we aimed to evaluate the correlation, over a short and restricted time, between dopaminergic neurodegeneration, astroglial activation, and MAPK expression (p-38/ERK) in old mice after acute MPTP intoxication. For that purpose, we analyzed two main different parameters in the nigrostriatal system: (i) Parkinsonism; and (ii) dynamic changes over time, both under aging conditions. The importance of using old mice lies on the implication of declined processes associated with aging, resulting to the description of being the main risk factor for PD (Collier et al., 2017). An inherent mechanism that contributes to this deleterious effect is the so-called inflammaging. It is a concept that involves chronic inflammatory mechanisms that form part of the complex adaptive response throughout the lifespan (Calabrese et al., 2018). Thereby, it is proposed as the principal process underlying age-related disorders like PD.

Specifically, it is highly reported that neuroinflammation is mediated by glial cells and, enhanced by aging processes, is key in the progression of dopaminergic neurodegeneration in PD (Stojkovska et al., 2015). However, it is still unclear how neurodegeneration and glial activation are related over time. We decided to perform the study in a temporary range from 4 to 48 h after an acute administration regimen of MPTP to observe the primary effects produced by the intoxication in old mice.

Our results show that both dopaminergic neuronal death and glial response start to be significantly increased in the striatum at 24 h after MPTP intoxication compared with that in control animals. These data are supported by observations in immunostained coronal brain sections from control and MPTP mice 48 h after MPTP administration (Figure 6). A decrease of TH expression in dopaminergic neurons and an increase of both astroglial and microglial cells in MPTP mice at 48 h after MPTP intoxication can be seen in both SNpc and striatum (Figure 6). Moreover, these results are in line with the observations of retrograde degeneration from the striatal terminal to dopaminergic neurons in the VM of rodents after MPTP intoxication. Contrarily, in humans and non-human primates, the degeneration occurs from dopaminergic neurons to striatal terminals (Dauer and Przedborski, 2003).

Moreover, both the phosphorylated forms of p38 and ERK expressions are altered in the aged brains (Zhen et al., 1999). In this study, it is shown that the phospho-p38 levels remain high and constant at 4 h after intoxication in the Parkinsonian animals. In the striatum, the expression of phospho-p38 is increased at 4 h after MPTP intoxication and becomes significant at 48 h compared to that of the control groups. On the other hand, no significant differences were observed in the activation of ERK both in the SNpc and in the striatum. Thus, based on these data and the idea that the p38 MAPK pathway contributes to neuroinflammation through glial cells (Kim and Choi, 2015), we point out that the processes related to cell death and inflammation respond faster to the stimulus produced by MPTP in the striatum than in the VM, whose significant effect can be observed 24 h after the intoxication.

These results are especially interesting because they show the timeline of events related to neuronal death and inflammation along with two related molecules in these processes. Different studies published evidence that p38 participates in neuronal death and the production of NO, together with its subproduct MK2 in glial cells (Thomas et al., 2008).

Moreover, regarding the published data using the MPTP model, the selective activation of p38 MAPK upstream of NF-kB in the SNpc neurons of mice treated with MPTP has been described as one that can promote neurodegeneration processes (Karunakaran and Ravindranath, 2009), and TNF-α was also identified as an early upstream activator of the ASK1-MAPK death signaling pathway after MPTP intoxication (Ray et al., 2015). IL-32b exaggerated the MPTP-mediated activation of p38 MAPK and JNK pathways, which have been shown to be involved in MPTP neurotoxicity (Jung et al., 2017). In fact, a new hypothesis for PD pathogenesis that points out the role of p38 MAPK pathway in the modulation of autophagy in cell death processes has recently been proposed (Obergasteiger et al., 2018).

In conclusion, we want to highlight the need to increase the studies focused on the MAPK pathway with the aim of developing therapeutic strategies applied at the right time point. In this way, we can delay the decline processes associated with aging, such as inflammaging, and, consequently, neuronal degeneration in PD patients.

## Data Availability Statement

All datasets generated for this study are included in the article.

## Ethics Statement

The animal study was reviewed and approved by the Institutional Committee on Animal Ethics of the University of Murcia (REGA ES300305440012).

## Author Contributions

AG-M carried out the postmortem experiments. LC-B, PG-S, CS-R, and VI performed the immunohistochemistry experiments and participated in the *in vivo* procedures and treatments. MH, EF-V, and AG-M designed the study and worked in the manuscript preparation. MH, EF-V, HS, and AG-M discussed the results. All the authors read and approved the final manuscript.

## Conflict of Interest

The authors declare that the research was conducted in the absence of any commercial or financial relationships that could be construed as a potential conflict of interest.
